# Medical AI Use Intention Among Medical Students and Faculty and Its Associations With AI Literacy, Perceived Benefits, and Risks: Cross-Sectional Survey Study

**DOI:** 10.2196/105032

**Published:** 2026-09-24

**Authors:** Bomyee Lee, Gwanwook Bang, Su Jin Chae

**Affiliations:** 1 Department of Medical Education, University of Ulsan College of Medicine, 30 Badeurae 1-gil, Dong-gu, Ulsan, 44033, Republic of Korea, +82-52-701-3715; 2 Department of Medical Education, Chosun University College of Medicine, Gwangju, Republic of Korea

**Keywords:** artificial intelligence, AI, AI use intention, AI literacy, perceived benefits, perceived risks, medical education, technology acceptance

## Abstract

**Background:**

AI is being increasingly integrated into health care and medical education. Although AI literacy is considered an essential competency for future physicians and educators, limited evidence exists regarding how perceived benefits, perceived risks, and AI literacy jointly influence AI use intention among different learner groups.

**Objective:**

This study examined the factors associated with AI use intention among medical students and faculty members and investigated whether the relationships between AI literacy and AI use intention differed according to learner group.

**Methods:**

A cross-sectional self-report survey was conducted among 141 medical students and 94 faculty members (N=235) at a single South Korean medical school. Group-specific linear regression models were estimated for descriptive purposes. To directly test whether associations differed between groups, a pooled regression model included group-by-predictor interaction terms for perceived benefits, perceived risks, AI literacy, and AI use frequency. HC3 heteroscedasticity–robust SEs were used for primary inference, and ordinal logistic regression was conducted as a sensitivity analysis because the 5-point intention outcome showed a marked ceiling effect.

**Results:**

Perceived benefits were positively associated with AI use intention in both groups. In the pooled HC3-robust model, the group-by–AI literacy interaction was significant (*B*=0.361, 95% CI 0.083-0.638; *P*=.01), whereas the interactions for perceived benefits, perceived risks, and AI use frequency were not significant. The group-by–AI literacy interaction remained significant in ordinal logistic regression (odds ratio 2.48, 95% CI 1.24-4.98; *P*=.01). In subgroup models, the negative adjusted AI literacy coefficient among students was model dependent, and the positive perceived risk coefficient was not robust to HC3 inference. Among faculty members, AI use frequency remained positively associated with use intention using both HC3 linear regression (*B*=0.137, 95% CI 0.025-0.249; *P*=.02) and ordinal logistic regression (*P*=.02).

**Conclusions:**

Perceived benefits were consistently associated with stronger AI use intention. The adjusted association between AI literacy and use intention differed between students and faculty, although the subgroup-specific student coefficient was sensitive to model specification. These findings provide exploratory evidence that learner context may shape the relationship between AI literacy and broad self-reported AI use intention; they do not establish causal effects or the effectiveness of specific curricular strategies.

## Introduction

AI is rapidly transforming health care by supporting clinical decision-making, diagnosis, treatment planning, and health care management. Recent advances in generative AI have further expanded its applications in medical education, including personalized learning, clinical reasoning, assessment, and feedback. As AI becomes increasingly integrated into health care, physicians are expected to use AI not merely as a technical tool but also as a collaborative partner in clinical practice. Accordingly, medical schools are challenged to prepare future physicians and educators who can critically evaluate AI-generated information, recognize its limitations, and integrate it appropriately into clinical decision-making and lifelong learning [1-5].

AI literacy has therefore emerged as an essential competency in medical education. AI literacy encompasses the knowledge, skills, and attitudes required to access, understand, critically evaluate, and appropriately apply AI technologies in professional practice [6-9]. International organizations, including the World Medical Association, have emphasized that physicians should develop competencies enabling them to use AI responsibly while maintaining professional accountability [6]. In parallel, many medical schools have begun introducing AI-related curricula and faculty development programs to prepare learners for AI-supported health care environments [7-9]. However, acquiring AI knowledge alone may not be sufficient to encourage meaningful AI adoption in educational or clinical settings.

Previous studies examining AI acceptance among health care professionals and medical students have consistently demonstrated that perceived usefulness or perceived benefits are among the strongest determinants of technology acceptance [10-14]. According to the technology acceptance model (TAM) and the unified theory of acceptance and use of technology (UTAUT), individuals are more likely to adopt a technology when they believe that it will improve their performance or productivity [13,14]. Conversely, perceived risks—including concerns regarding diagnostic errors, ethical issues, privacy, and professional responsibility—may reduce willingness to adopt AI, although previous findings have been inconsistent across health care settings and participant groups [10,15-17].

In the present study, perceived benefits were used as a context-specific umbrella construct corresponding conceptually to perceived usefulness in the TAM and performance expectancy in the UTAUT. It encompassed more specific expectations that AI may improve efficiency, diagnostic or clinical accuracy, and decision support. Perceived risks represented a distinct evaluative belief concerning potential harm, including impaired judgment, AI-related error, and privacy concerns, rather than the simple inverse of perceived benefits. AI literacy was conceptualized separately as a self-reported capability to access, understand, evaluate, and apply AI-related information, not as an acceptance belief. The learner group was examined as a contextual moderator reflecting differences in professional responsibility, experience, and opportunities for authentic AI use. Accordingly, this study did not test a complete causal TAM or UTAUT model; it used an exploratory association framework to examine the unique and potentially group-dependent contributions of these constructs to broad AI use intention.

Compared with perceived benefits and risks, the educational role of AI literacy remains less clear. AI literacy is generally regarded as a desirable educational outcome because it improves users’ ability to understand AI technologies and critically evaluate AI-generated information. However, greater AI literacy may also increase awareness of AI limitations, uncertainty, and ethical concerns, potentially leading to more cautious attitudes toward AI use. Previous studies have therefore reported inconsistent relationships between AI literacy and AI acceptance, suggesting that its influence may depend on learners’ educational experiences and professional contexts rather than representing a uniformly positive determinant of AI use intention [18,19].

An important gap remains in the existing literature. Most previous studies have investigated medical students or health care professionals separately, making it difficult to determine whether the factors associated with AI use intention operate similarly across learner groups [10-12,18,19]. Medical students and faculty members differ substantially in clinical experience, educational responsibilities, opportunities to apply AI in practice, and expectations regarding patient care. These contextual differences may influence how perceived benefits, perceived risks, and AI literacy contribute to AI use intention. Understanding these differences is important because educational strategies that are effective for one learner group may not be equally effective for another.

Therefore, this study examined the factors associated with AI use intention among medical students and faculty members. Specifically, we investigated the relative contributions of perceived benefits, perceived risks, and AI literacy to AI use intention and examined whether the relationship between AI literacy and AI use intention differed according to learner group.

## Methods

### Study Design

This cross-sectional survey was conducted between January and February 2026 at the University of Ulsan College of Medicine to examine factors associated with AI use intention among medical students and faculty members. Specifically, this study compared AI-related characteristics, AI perceptions, AI literacy, and AI use intention between the 2 groups and identified factors independently associated with AI use intention within each group.

### Participants

Eligible participants included all enrolled second-year premedical students and first- through fourth-year medical students (n=200), as well as all full-time faculty members (n=532) at the University of Ulsan College of Medicine. First-year premedical students were excluded because the survey was conducted before their enrollment.

An invitation containing information about the study and a link to the anonymous web-based questionnaire was distributed through the institutional communication system. Participation was voluntary, and no incentives were provided.

A total of 235 participants completed the survey, including 141 (60%) medical students (response rate: 141/200, 70.5%) and 94 (40%) faculty members (response rate: 94/532, 17.7%). Only complete questionnaires were included in the final analyses. This online survey is reported in accordance with the CHERRIES (Checklist for Reporting Results of Internet E-Surveys) guidelines (Checklist 1).

### Ethical Considerations

This study was approved by the Institutional Review Board of Asan Medical Center (S2025-2554-0001). Before accessing the questionnaire, all participants received information regarding the study purpose, voluntary participation, confidentiality, and data protection. Electronic informed consent was obtained before participation.

The survey was conducted anonymously, and no personally identifiable information was collected. All data were stored in deidentified form and used exclusively for research purposes.

### Measures

#### Overview

The questionnaire included items assessing demographic and AI-related characteristics, AI perceptions, AI use intention, and AI literacy. To establish content validity, 2 experts in medical education independently reviewed all questionnaire items for clarity, relevance, and appropriateness. The questionnaire was revised based on their feedback before finalization.

#### Demographic and AI-Related Characteristics

Demographic variables included sex, age, medical specialty (or intended career path for students), and previous experience with AI education. AI-related characteristics included familiarity with AI, self-rated AI knowledge, and frequency of AI use.

#### AI Perceptions

Items assessing perceived benefits, perceived risks, and AI use intention were adapted from the TAM [13], the UTAUT [14], and previous studies on AI acceptance in medicine [10,20].

Perceived benefits were measured using 6 items assessing expectations regarding AI-assisted diagnosis and treatment, improved efficiency, and enhanced clinical accuracy. Perceived risks were measured using 3 items assessing concerns related to impaired clinical judgment, potential diagnostic errors, and privacy issues. Item-level descriptive statistics, corrected item-total correlations, and Cronbach α values if each item was deleted are provided in Table S1 in Multimedia Appendix 1.

AI use intention was measured using a single item assessing participants’ intention to use AI in future education, clinical practice, and research. All items were rated on a 5-point Likert scale, with higher scores indicating stronger agreement (Multimedia Appendix 2).

#### AI Literacy

AI literacy was measured using a 10-item instrument developed based on the integrated health literacy model proposed by Sørensen et al [21] and adapted to AI competencies described by Miao et al [22]. The instrument consisted of 4 domains: access (2 items), understanding (2 items), judgment (3 items), and application (3 items). The 4 domains assess participants’ ability to identify reliable AI information sources, understand AI-related concepts, critically evaluate AI-generated information, and appropriately apply AI in educational and professional contexts. All items were rated on a 5-point Likert scale, with higher scores indicating greater AI literacy (Multimedia Appendix 3).

### Statistical Analysis

Statistical analyses were performed using Jamovi (version 2.7.24). Continuous variables are presented as means and SDs, whereas categorical variables are presented as frequencies and percentages. The internal consistency of all multi-item scales was evaluated using Cronbach α coefficients, with values greater than 0.70 considered acceptable. Skewness and kurtosis were examined to assess the assumption of univariate normality.

Baseline characteristics were compared between medical students and faculty members using independent-sample 2-tailed *t* tests for continuous variables and chi-square tests for categorical variables. Homogeneity of variances was assessed using the Levene test, and the Welch *t* test was applied when the assumption of equal variances was violated.

Unadjusted between-group comparisons were considered the primary descriptive comparisons. Exploratory analyses of covariance additionally adjusted for sex, age, and previous AI education because these characteristics differed between groups. However, age was strongly associated with learner group, and the age distributions of students and faculty had limited overlap. Therefore, age-adjusted group estimates were treated as sensitivity analyses and were not interpreted as representing a causal or directly comparable student-faculty contrast.

Pearson correlation analyses were conducted separately for medical students and faculty members. Group-specific multiple linear regression models were estimated to describe associations with AI use intention. To directly test whether predictor associations differed by participant group, a pooled regression model included participant group and group-by-predictor interaction terms for perceived benefits, perceived risks, total AI literacy, and AI use frequency. Continuous predictors were mean centered before interaction terms were constructed. The pooled model additionally included AI knowledge, AI familiarity, sex, age, and previous AI education. Because AI use intention was measured on a bounded 5-point scale and showed a marked ceiling effect, HC3 heteroscedasticity–robust SEs were used for primary inferential interpretation. Ordinal logistic regression was conducted as a sensitivity analysis using the same predictor set. For this analysis, perceived benefits, perceived risks, total AI literacy, and AI use frequency were standardized before constructing the group-by-predictor interaction terms; thus, the corresponding odds ratios represent associations per 1-SD increase. The proportional odds assumption was also assessed. Complete ordinal logistic regression results, including main effects, interaction terms, threshold estimates, model fit statistics, and assessment of the proportional odds assumption, are provided in Table S2 in Multimedia Appendix 1. Additional sensitivity analyses examined reduced predictor sets to evaluate the stability of the student AI literacy coefficient. Multicollinearity was assessed using variance inflation factors and tolerance statistics. Results are reported as unstandardized coefficients; standardized coefficients where applicable; odds ratios for ordinal logistic regression; 95% CIs; and 2-sided *P* values, with *P*<.05 considered statistically significant.

## Results

### Demographic Characteristics of the Participants

A total of 235 participants completed the survey, comprising 141 (60%) medical students and 94 (40%) faculty members (Table 1). Among the students, 58.2% (82/141) were male, and the mean age was 23.6 (SD 1.87) years. Second-year premedical students constituted the largest academic year subgroup (38/141, 27.0%). Regarding intended career paths, surgery was the most frequently selected option (53/141, 37.6%).

Among the faculty members, 71.3% (67/94) were male, and the mean age was 48.9 (SD 7.58) years. Half (47/94, 50%) of the faculty participants were professors. Regarding medical specialties, other clinical specialties represented the largest category (45/94, 47.9%). Their mean professional experience was 17.3 (SD 8.19) years.

**Table 1.** Participant characteristics.

Characteristics and categories	Students (n=141)	Faculty (n=94)
Sex, n (%)		
Male	82 (58.2)	67 (71.3)
Female	59 (41.8)	27 (28.7)
Age (y), mean (SD)	23.6 (1.87)	48.9 (7.58)
Academic year, n (%)		
Premedical year 2	38 (27.0)	—^a^
Medical year 1	35 (24.8)	—
Medical year 2	21 (14.9)	—
Medical year 3	31 (22.0)	—
Medical year 4	16 (11.3)	—
Academic position, n (%)		
Professor	—	47 (50)
Associate professor	—	27 (28.7)
Assistant professor	—	19 (20.2)
Nontenure position	—	1 (1.1)
Professional experience (y), mean (SD)	—	17.3 (8.19)
Intended career path (students) or specialty (faculty), n (%)		
Internal medicine	36 (25.5)	22 (23.4)
Surgery	53 (37.6)	16 (17.0)
Other clinical specialties	36 (25.5)	45 (47.9)
Research or nonclinical field	3 (2.1)	11 (11.7)
Undecided	13 (9.2)	—

a Not applicable.

### Comparison of Demographic and AI-Related Characteristics Between Students and Faculty

In the unadjusted comparisons, significant between-group differences were observed for sex (*χ*^2^_1_=4.2; *P*=.04), age (*t*_100.6_=−31.7; *P*<.001), and previous AI education (*χ*^2^_1_=18.2; *P*<.001; Table 2). Faculty members included a higher proportion of male individuals, whereas students reported greater previous AI education experience. Because age was strongly confounded with learner group and the observed age ranges had limited overlap, the covariate-adjusted estimates that follow are presented as exploratory sensitivity analyses and should not be interpreted as comparisons of otherwise equivalent students and faculty members.

In the exploratory covariate-adjusted analysis, faculty members reported more frequent AI use than students (adjusted mean 4.13, SE 0.224 vs 3.30, SE 0.159; *F*_1,230_=5.550; *P*=.02). No adjusted differences were observed in AI knowledge level (*F*_1,230_=0.132; *P*=.72) or AI familiarity (*F*_1,230_=0.304; *P*=.58). These adjusted findings should be interpreted cautiously because of the limited age overlap between groups.

**Table 2.** Comparison of demographic and AI-related characteristics between medical students and faculty members.

Characteristics	Students (n=141)	Faculty (n=94)	Statistic (*df*)	*P* value
Male sex, n (%)	82 (58.2)	67 (71.3)	*χ*^2^=4.2 (1)	.04
Age (y), mean (SD)	23.6 (1.87)	48.9 (7.58)	*t*=–31.7 (100.6)	<.001
Previous AI education, n (%)	98 (69.5)	39 (41.5)	*χ*^2^=18.2 (1)	<.001
AI use frequency^a^ (possible range 1-5), adjusted mean (SE)	3.30 (0.159)	4.13 (0.224)	*F*=5.550 (1,230)	.02
AI knowledge^a^ (possible range 1-5), adjusted mean (SE)	2.53 (0.131)	2.64 (0.185)	*F*=0.132 (1,230)	.72
AI familiarity^a^ (possible range 1-5), adjusted mean (SE)	2.86 (0.150)	3.04 (0.212)	*F*=0.304 (1,230)	.58

a Adjusted means from analysis of covariance controlling for sex, age, and previous AI education.

### Reliability of the Study Instruments

The AI perception and AI literacy instruments demonstrated acceptable internal consistency in the present sample. The Cronbach α was 0.884 for the perceived benefits subscale and 0.617 for the perceived risks subscale. For the AI literacy instrument, Cronbach α values ranged from 0.726 to 0.847 across the 4 domains, and the overall scale demonstrated excellent internal consistency (Cronbach α=0.899).

### Between-Group Differences in AI Perceptions and AI Literacy

In exploratory analysis of covariance sensitivity analyses adjusting for sex, age, and previous AI education, no statistically significant differences were observed between medical students and faculty members in perceived benefits, perceived risks, AI use intention, or any AI literacy domain (*P*>.05 in all cases; Table 3). Given the strong association between age and learner group and limited age overlap, these adjusted means are descriptive sensitivity estimates rather than evidence of equivalent student-faculty populations. Item-level analysis of the perceived-risk scale in the full sample showed mean scores of 3.65 (SD 1.10), 3.81 (SD 0.91), and 3.93 (SD 0.94) for impaired clinical judgment, potential diagnostic errors, and privacy concerns, respectively; corrected item-total correlations ranged from 0.391 to 0.455.; detailed item-level results are provided in Table S1 in Multimedia Appendix 1.

**Table 3.** Adjusted comparisons of AI perceptions and AI literacy between medical students and faculty members. Adjusted means were estimated in an exploratory analysis of covariance sensitivity analysis controlling for sex, age, and previous AI education. Because age was strongly associated with learner group and age distributions had limited overlap, adjusted group estimates should be interpreted cautiously.

Categories and variables	Students, mean (SE)	Faculty, mean (SE)	*F* test (*df*)	*P* value
AI perception				
Perceived benefits (possible range 1-5)	3.94 (0.101)	4.02 (0.142)	0.127 (1,230)	.72
Perceived risks (possible range 1-5)	3.90 (0.117)	3.63 (0.165)	1.111 (1,230)	.29
AI use intention (possible range 1-5)	4.30 (0.109)	4.56 (0.155)	1.068 (1,230)	.30
AI literacy				
Access (possible range 1-5)	3.91 (0.126)	4.27 (0.178)	1.674 (1,230)	.20
Understanding (possible range 1-5)	3.97 (0.110)	4.29 (0.156)	1.748 (1,230)	.19
Judgment (possible range 1-5)	3.71 (0.113)	3.96 (0.159)	0.958 (1,230)	.33
Application (possible range 1-5)	3.89 (0.105)	3.89 (0.148)	<0.001 (1,230)	.98
Total (possible range 1-5)	3.85 (0.094)	4.07 (0.132)	1.043 (1,230)	.31

### Correlation Analysis

Correlation analyses were conducted separately for medical students and faculty members (Tables 4 and 5). Among medical students, perceived benefits showed a strong positive correlation with AI use intention (*r*=0.660; *P*<.001), whereas total AI literacy was weakly and nonsignificantly associated with AI use intention (*r*=0.155; *P*=.07). Perceived risks demonstrated a weak positive correlation with AI use intention (*r*=0.293; *P*<.001).

Among faculty members, both perceived benefits (*r*=0.588; *P*<.001) and total AI literacy (*r*=0.408; *P*<.001) were positively correlated with AI use intention, whereas perceived risks were not significantly associated with AI use intention (*r*=0.140; *P*=.18).

**Table 4.** Pearson correlations among AI perceptions, AI literacy, and AI use intention in medical students (n=141).

	Perceived benefits	Perceived risks	Total AI literacy	AI use intention
Perceived benefits	1	0.238^a^	0.295	0.660
Perceived risks	0.238^a^	1	0.427	0.293
Total AI literacy	0.295^b^	0.427^b^	1	0.155
AI use intention	0.660^b^	0.293^b^	0.155	1

a *P*<.01.

b *P*<.001.

**Table 5.** Pearson correlations among AI perceptions, AI literacy, and AI use intention in faculty members (n=94).

	Perceived benefits	Perceived risks	Total AI literacy	AI use intention
Perceived benefits	1	0.170	0.332	0.588
Perceived risks	0.170	1	0.005	0.140
Total AI literacy	0.332^a^	0.005	1	0.408
AI use intention	0.588^b^	0.140	0.408^b^	1

a *P*<.01.

b *P*<.001.

### Factors Associated With AI Use Intention

Group-specific multiple linear regression models retaining the full covariate set (perceived benefits, perceived risks, AI literacy, AI use frequency, AI knowledge, AI familiarity, sex, age, and previous AI education) were estimated for students and faculty members (Table 6). HC3 heteroscedasticity–robust SEs were used as the primary basis for inference because the bounded intention outcome showed a marked ceiling effect.

Among faculty members, perceived benefits were positively associated with AI use intention (*B*=0.529, HC3 95% CI 0.288-0.771; standardized β=0.503; *P*<.001). AI use frequency also remained positively associated with intention (*B*=0.137, HC3 95% CI 0.025-0.249; standardized β=0.257; *P*=.02). AI literacy was positive in the conventional linear model but marginal with HC3 inference (*B*=0.221, HC3 95% CI −0.018 to 0.460; *P*=.07).

**Table 6.** Multiple linear regression models predicting AI use intention among medical students and faculty members.^a^

Predictors	Students (n=141)^b^		Faculty (n=94)^c^	
	*B* (HC3 95% CI)	β	*B* (HC3 95% CI)	β
Perceived benefits	0.665^d^ (0.503 to 0.828)	0.590	0.529^d^ (0.288 to 0.771)	0.503
Perceived risks	0.187 (−0.037 to 0.411)	0.192	0.056 (−0.082 to 0.194)	0.060
AI literacy	−0.197^e^ (−0.382 to −0.012)	−0.163	0.221 (−0.018 to 0.460)	0.200
AI use frequency	0.063 (−0.074 to 0.200)	0.079	0.137^e^ (0.025 to 0.249)	0.257
AI knowledge level	0.062 (−0.067 to 0.191)	0.078	−0.048 (−0.278 to 0.182)	−0.054
Familiarity	0.063 (−0.077 to 0.202)	0.083	−0.097 (−0.263 to 0.069)	−0.140
Sex (female)	−0.120 (−0.318 to 0.078)	−0.082	−0.069 (−0.349 to 0.211)	−0.047
Age	−0.017 (−0.073 to 0.038)	−0.044	0.003 (−0.015 to 0.022)	0.038
AI education experience	0.096 (−0.145 to 0.336)	0.061	0.106 (−0.164 to 0.375)	0.078

a *B* indicates the unstandardized regression coefficient, and β indicates the standardized coefficient from the corresponding ordinary least squares model. The 95% CIs and *P* values were estimated using HC3 heteroscedasticity–robust SEs. All variance inflation factors were below 2.0, indicating no problematic multicollinearity.

b *R*2=0.510; adjusted *R*2=0.476; *F*_9,131_=15.14; *P*<.001.

c *R*2=0.452; adjusted *R*2=0.393; *F*_9,84_=7.69; *P*<.001.

d *P*<.001

e *P*<.05.

### Pooled Interaction and Sensitivity Analyses

In the pooled HC3-robust regression model (N=235), the group-by–AI literacy interaction was statistically significant (*B*=0.361, 95% CI 0.083-0.638; *P*=.01), indicating that the adjusted association between AI literacy and use intention differed between students and faculty members. The interactions for perceived benefits, perceived risks, and AI use frequency were not significant (Table 7). The model explained 47.0% of the variance (adjusted *R*^2^=0.436; *F*_14,220_=11.88; *P*<.001).

AI use intention showed a marked ceiling effect: 55.3% (130/235) of participants selected “strongly agree” (score 5), and 36.6% (86/235) selected “agree” (score 4). In ordinal logistic sensitivity analyses, the group-by–AI literacy interaction remained significant (odds ratio 2.48, 95% CI 1.24-4.98; *P*=.01), whereas the other interactions remained nonsignificant. Complete ordinal logistic regression results, including main effects, interaction terms, threshold estimates, model fit statistics, and assessment of the proportional odds assumption, are provided in Table S2 in Multimedia Appendix 1. The student AI literacy coefficient was not stable across reduced linear models, and the positive student perceived risk coefficient was not significant with HC3 robust inference. These findings support limiting the principal group difference claim to the AI literacy interaction and interpreting subgroup coefficients cautiously.

**Table 7.** Group-by-predictor interactions in pooled HC3 linear and ordinal logistic regression models.^a^

Interaction	*B* (HC3 95% CI)	*P* value	Ordinal OR^b^ (95% CI)	*P* value
Group × perceived benefits	−0.172 (−0.452 to 0.107)	.23	0.73 (0.36 to 1.49)	.39
Group × perceived risks	−0.132 (−0.405 to 0.140)	.34	0.73 (0.37 to 1.44)	.36
Group × AI literacy	0.361 (0.083 to 0.638)	.01	2.48 (1.24 to 4.98)	.01
Group × AI use frequency	−0.002 (−0.151 to 0.148)	.98	0.98 (0.48 to 1.96)	.94

a *B* indicates the unstandardized regression coefficient from the pooled linear regression model estimated using HC3 heteroscedasticity–robust SEs. *P* values were estimated using HC3 heteroscedasticity–robust SEs for the linear regression model.

b OR: odds ratio from the ordinal logistic regression model.

## Discussion

### Principal Findings

This study examined factors associated with broad self-reported AI use intention among medical students and faculty members within a single medical school. Three principal findings emerged.

First, perceived benefits were consistently and strongly associated with AI use intention in both learner groups. This association remained robust across the primary regression analyses and sensitivity analyses using HC3 heteroscedasticity–robust SEs. These findings suggest that participants who perceived AI as beneficial for education, research, or clinical practice were more likely to report intentions to use AI regardless of learner group.

Second, the pooled interaction analysis demonstrated that the adjusted association between AI literacy and AI use intention differed significantly between medical students and faculty members. Importantly, this conclusion was based on direct statistical testing of the interaction rather than comparison of separate subgroup regressions. However, the subgroup-specific pattern should be interpreted cautiously. Among students, the zero-order association between AI literacy and use intention was weak and positive, whereas the adjusted regression coefficient became negative only after controlling for correlated AI-related variables and varied across reduced models. This pattern is compatible with suppression or shared variance rather than a stable inverse relationship. In contrast, among faculty members, AI literacy showed a positive association with AI use intention that was generally consistent across analyses.

Third, although perceived risks appeared positively associated with AI use intention among students in the conventional regression model, this association was attenuated and no longer statistically significant when HC3-robust SEs were applied. Moreover, the pooled interaction model did not demonstrate a significant difference between students and faculty members for perceived risks. Accordingly, this finding should be regarded as exploratory rather than evidence that greater awareness of AI-related risks increases willingness to use AI.

### Comparison With Previous Studies

The finding that perceived benefits represented the strongest correlate of AI use intention is consistent with those of previous studies examining technology acceptance in health care. The TAM and UTAUT consistently identify perceived usefulness or performance expectancy as the principal determinant of behavioral intention. Similarly, recent studies involving physicians, medical students, and other health care professionals have reported that expected educational or clinical benefits are the most influential predictors of AI acceptance. Our findings extend this literature by demonstrating that perceived benefits remained the dominant correlate of AI use intention even after accounting for AI literacy, perceived risks, AI familiarity, AI knowledge, and previous AI education.

The relationship between AI literacy and AI use intention was more complex. Previous studies have generally assumed that greater AI literacy facilitates AI adoption because users become more confident in understanding and applying AI technologies. However, there is emerging evidence also suggesting that greater AI literacy may increase awareness of AI limitations, uncertainty, ethical concerns, and the need for critical evaluation. Rather than demonstrating that AI literacy has uniformly positive or negative effects, our findings suggest that its association with AI use intention may depend on learners’ educational and professional contexts. Faculty members who routinely integrate AI into teaching, research, or clinical work may experience AI literacy primarily as a practical competency, whereas students may simultaneously acquire greater awareness of both the opportunities and limitations of AI during training. Because the adjusted student coefficient was sensitive to model specification, these findings should be interpreted as evidence of contextual variation rather than evidence that greater AI literacy reduces students’ willingness to use AI.

Our findings also highlight the importance of distinguishing between broad AI use and specific AI applications. The present questionnaire assessed general intention to use AI across education, research, and clinical practice rather than focusing on a particular AI technology such as generative AI or clinical decision support systems. These contexts differ substantially with respect to task complexity, responsibility, explainability requirements, governance, and acceptable levels of risk. Recent studies suggest that trust, governance, institutional policies, and clinical context substantially influence AI adoption in health care. For example, a 2026 survey of health care practitioners found that trust and perceived risk jointly shaped AI acceptance and readiness, with trust functioning as a construct distinct from perceived benefit or risk alone [23], and work on health chatbot adoption has shown that perceived risk lowers trust both directly and indirectly through perceived usefulness and self-efficacy [24]. Separately, studies of generative AI integration into medical curricula indicate that adoption is shaped substantially by institutional governance, including policy on permitted use and equitable platform access, in addition to individual perceptions of benefit or risk [25]. Because trust and governance constructs were not directly measured in the present study, future investigations should incorporate them explicitly, distinguish among different categories of AI technologies, and examine how determinants of AI acceptance vary across educational and clinical settings.

### Educational Implications

Although the present findings are exploratory, they suggest several implications for AI education in medical schools. The significant interaction between learner group and AI literacy indicates that identical educational approaches may not address the needs of students and faculty members equally. Future educational interventions for students may benefit from emphasizing supervised AI application, critical appraisal of AI-generated information, appropriate calibration of trust, and responsible clinical reasoning rather than focusing exclusively on conceptual knowledge. For faculty members, educational programs may place greater emphasis on integrating AI into authentic teaching, research, and clinical activities while supporting confident and appropriate implementation.

Importantly, these implications should be regarded as educational hypotheses rather than evidence-based curricular recommendations. Because this study did not evaluate educational interventions, curriculum design, or subsequent AI use behavior, further longitudinal and intervention studies are needed before specific educational strategies can be recommended.

### Limitations

Several limitations should be acknowledged.

First, participants were recruited from a single medical school, and the faculty response rate was relatively low. Consequently, self-selection bias cannot be excluded, and the findings may not be generalizable to other institutions or health care settings.

Second, the cross-sectional design and reliance on self-reported questionnaire data preclude causal inference. The observed associations cannot determine whether AI use frequency increases AI use intention, whether stronger intentions lead to more frequent AI use, or whether both are influenced by other unmeasured factors.

Third, AI use intention was assessed using a single broad item encompassing education, research, and clinical practice. Although this approach captured participants’ overall intention to use AI, it could not distinguish among different AI technologies or application contexts and did not permit assessment of measurement error. Future studies should use validated multi-item measures and separately examine intentions related to generative AI, clinical decision support systems, and other AI applications.

Fourth, although the AI literacy instrument was adapted from established theoretical frameworks and demonstrated acceptable internal consistency, additional psychometric evaluation—including exploratory or confirmatory factor analysis, construct validation, and measurement invariance across learner groups—was beyond the scope of the present study. Future research should further validate the measurement properties of this instrument in larger and more diverse samples.

Finally, the perceived risk subscale demonstrated relatively limited internal consistency and combined concerns related to clinical judgment, diagnostic errors, and privacy. Furthermore, the positive association between perceived risks and AI use intention among students was not robust in HC3 sensitivity analyses, and the corresponding interaction effect was not statistically significant. Consequently, findings related to perceived risks should be interpreted cautiously until replicated in future studies using more refined measures of AI-related risk.

### Conclusions

Perceived benefits were consistently associated with stronger broad self-reported AI use intention among both medical students and faculty members. The association between AI literacy and AI use intention differed according to learner group, although the underlying mechanism remains uncertain. These findings provide exploratory evidence that learner context may shape AI use intention and highlight the need for multicenter longitudinal studies using validated measures to better understand AI adoption in medical education.

## Supplementary material

10.2196/105032 Multimedia Appendix 1 Item-level descriptive statistics for the perceived-risk scale and complete ordinal logistic regression model predicting AI use intention in the pooled sample.

10.2196/105032 Multimedia Appendix 2 AI perception survey instrument.

10.2196/105032 Multimedia Appendix 3 AI literacy survey instrument.

10.2196/105032 Checklist 1 CHERRIES checklist.

## Abbreviations

CHERRIES: Checklist for Reporting Results of Internet E-Surveys
TAM: technology acceptance model
UTAUT: unified theory of acceptance and use of technology
