# Supplementary material for: Medical AI Use Intention Among Medical Students and Faculty and Its Associations With AI Literacy, Perceived Benefits, and Risks: Cross-Sectional Survey Study
Source: JMIR Med Educ. 2026 Sep 24;12:e105032. doi: 10.2196/105032 (PMC13606327; doi:10.2196/105032)
Supplement: Supplementary file 1 [file mededu-v12-e105032-s001.docx]

**Multimedia Appendix 1**

**Table S1. Item-level descriptive statistics for the perceived-risk scale (N=235)**

| Item | Construct assessed | Mean (SD) | Corrected item-total correlation |
| --- | --- | --- | --- |
| Item 1 | Impaired clinical judgment | 3.65 (1.10) | 0.455 |
| Item 2 | Potential diagnostic errors | 3.81 (0.91) | 0.439 |
| Item 3 | Privacy concerns | 3.93 (0.94) | 0.391 |

*Note****.*** *Corrected item-total correlation refers to the Pearson correlation between each item and the sum of the remaining two items. The scale's overall internal consistency was Cronbach's α=.617. Cronbach's α if the respective item were deleted was .478 (Item 1), .503 (Item 2), and .564 (Item 3), indicating that no single item was disproportionately responsible for the scale's relatively low internal consistency. The item-level results should be interpreted in light of the scale's brevity and the conceptually distinct risk domains represented by the three items.*

**Table S2. Complete ordinal logistic regression model predicting AI use intention in the pooled sample (N=235)**

| **Predictor** | **OR** | **95% CI** | **P value** |
| --- | --- | --- | --- |
| Participant group (faculty vs students) | 1.56 | 0.27–9.04 | .618 |
| Perceived benefits | 6.35 | 3.85–10.48 | <.001 |
| Perceived risks | 1.83 | 1.17–2.85 | .008 |
| AI literacy | 0.70 | 0.45–1.09 | .116 |
| AI use frequency | 1.76 | 1.07–2.92 | .027 |
| AI knowledge level | 1.32 | 0.83–2.11 | .236 |
| AI familiarity | 0.86 | 0.57–1.31 | .480 |
| Sex (female vs male) | 0.48 | 0.25–0.92 | .028 |
| Age | 1.01 | 0.95–1.08 | .756 |
| Previous AI education | 1.33 | 0.68–2.60 | .403 |
| Group × perceived benefits | 0.73 | 0.36–1.49 | .392 |
| Group × perceived risks | 0.73 | 0.37–1.44 | .357 |
| Group × AI literacy | 2.48 | 1.24–4.98 | .010 |
| Group × AI use frequency | 0.98 | 0.48–1.96 | .944 |

**Threshold estimates**

| **Threshold** | **Estimate** | **95% CI** |
| --- | --- | --- |
| 1 \| 2 | −8.10 | −11.53 to −4.66 |
| 2 \| 3 | −6.42 | −9.26 to −3.57 |
| 3 \| 4 | −3.15 | −5.48 to −0.82 |
| 4 \| 5 | 0.46 | −1.81 to 2.74 |

**Model fit:** likelihood-ratio χ²(14)=143.51, P<.001; log likelihood=−149.64; AIC=335.29; McFadden pseudo-R²=.324.

*Note****.*** *OR indicates odds ratio; CI, confidence interval. Perceived benefits, perceived risks, AI literacy, and AI use frequency were standardized before construction of the group-by-predictor interaction terms; therefore, the corresponding ORs represent associations per 1-SD increase. Students and male participants were the reference categories for participant group and sex, respectively. Threshold estimates are reported on the logit scale. Because responses in the two lowest AI use intention categories were extremely sparse (n=1 and n=2, respectively), a conventional full-sample Brant/parallel-lines test could not be estimated reliably. As a supplementary diagnostic, equality of slopes across the two estimable upper cumulative logits was not rejected (χ²=6.73, df=14, P=.945).*
