# Supplementary material for: Medical AI Use Intention Among Medical Students and Faculty and Its Associations With AI Literacy, Perceived Benefits, and Risks: Cross-Sectional Survey Study
Source: JMIR Med Educ. 2026 Sep 24;12:e105032. doi: 10.2196/105032 (PMC13606327; doi:10.2196/105032)
Supplement: Supplementary file 2 [file mededu-v12-e105032-s002.docx]

**Multimedia Appendix 1**.

**Item Pool**

| **No.** | **Item (verbatim wording as administered to participants)** | **Response scale** |
| --- | --- | --- |
| **A. Perceived Benefits (6 items)** | | |
| B1 | I believe medical AI is helpful in clinical decision-making processes such as diagnosis, treatment, and prognosis. | 1–5 (5-point Likert) |
| B2 | I think medical AI can improve the accuracy of diagnosis and treatment planning in specific clinical situations. | 1–5 (5-point Likert) |
| B3 | I believe medical AI can contribute to improving work efficiency in clinical settings. | 1–5 (5-point Likert) |
| B4 | I think the outputs of medical AI can be trusted as a reference for clinical judgment. | 1–5 (5-point Likert) |
| B5 | I believe the reasoning provided by medical AI is sufficient to serve as a reference for clinical decision-making. | 1–5 (5-point Likert) |
| B6 | I think medical AI can help compensate for errors in human decision-making. | 1–5 (5-point Likert) |
| **B. Perceived Risks (3 items)** | | |
| R1 | I believe that increasing reliance on medical AI may weaken human judgment capabilities. | 1–5 (5-point Likert) |
| R2 | I think errors in medical AI have the potential to lead to diagnostic delays or inappropriate treatment. | 1–5 (5-point Likert) |
| R3 | I believe that personal information may not be adequately protected during the development and use of AI. | 1–5 (5-point Likert) |
| **C. AI Use Intention (1 item)** | | |
| I1 | I intend to use medical AI in my work, learning, and research in the future. | 1–5 (5-point Likert) |

**Response Anchors**

1 = Strongly disagree; 5 = Strongly agree (intermediate points 2–4 unlabeled on the original instrument).
