# Supplementary material for: Medical AI Use Intention Among Medical Students and Faculty and Its Associations With AI Literacy, Perceived Benefits, and Risks: Cross-Sectional Survey Study
Source: JMIR Med Educ. 2026 Sep 24;12:e105032. doi: 10.2196/105032 (PMC13606327; doi:10.2196/105032)
Supplement: Supplementary file 3 [file mededu-v12-e105032-s003.docx]

**Multimedia Appendix 2**

**Item Pool**

| **No.** | **Item (verbatim wording as administered to participants)** | **Response scale** |
| --- | --- | --- |
| **A. Access (2 items)** | | |
| A1 | I try to verify the source of medical and health information provided by medical AI. | 1–5 (5-point Likert) |
| A2 | I try to check the evidence or basis for the health information that medical AI presents. | 1–5 (5-point Likert) |
| **B. Understanding (2 items)** | | |
| U1 | I can understand the content of medical and health information provided by medical AI. | 1–5 (5-point Likert) |
| U2 | I can understand the warnings and precautions included in medical information provided by medical AI. | 1–5 (5-point Likert) |
| **C. Judgment (3 items)** | | |
| J1 | I can evaluate the reliability of medical information provided by medical AI based on its source and validity. | 1–5 (5-point Likert) |
| J2 | I can make judgments when the content presented by medical AI differs from existing clinical guidelines or expert opinion. | 1–5 (5-point Likert) |
| J3 | I can assess the limitations and potential errors of information provided by medical AI. | 1–5 (5-point Likert) |
| **D. Application (3 items)** | | |
| P1 | I can apply medical and health information provided by medical AI appropriately to specific situations. | 1–5 (5-point Likert) |
| P2 | When referring to information from medical AI, I consider privacy protection and ethical considerations. | 1–5 (5-point Likert) |
| P3 | I can recognize and manage potential risks that may arise when using AI-based medical health information. | 1–5 (5-point Likert) |

**Response Anchors**

1 = Strongly disagree; 5 = Strongly agree (intermediate points 2–4 unlabeled on the original instrument).
