# Supplementary material for: Medical AI Use Intention Among Medical Students and Faculty and Its Associations With AI Literacy, Perceived Benefits, and Risks: Cross-Sectional Survey Study
Source: JMIR Med Educ. 2026 Sep 24;12:e105032. doi: 10.2196/105032 (PMC13606327; doi:10.2196/105032)
Supplement: Supplementary file 4 [file mededu-v12-e105032-s004.docx]

# Multimedia Appendix 3. CHERRIES Checklist

Checklist for Reporting Results of Internet E-Surveys (CHERRIES)

| CHERRIES item | Description | Addressed in manuscript |
| --- | --- | --- |
| Design | Cross-sectional web-based survey | Methods – Study Design |
| IRB approval | Approved by the Institutional Review Board of Asan Medical Center (IRB No. S2025-2554-0001) | Methods – Ethical Considerations |
| Informed consent | Participants received study information and provided electronic informed consent before accessing the questionnaire. | Methods – Ethical Considerations |
| Data protection | Anonymous survey; no personally identifiable information collected; de-identified data stored securely. | Methods – Ethical Considerations |
| Questionnaire development | Questionnaire reviewed independently by two medical education experts and revised before finalization. | Methods – Measures |
| Recruitment process | Invitation distributed through the institutional communication system. | Methods – Participants |
| Eligibility criteria | Eligible participants included enrolled medical students and full-time faculty members. | Methods – Participants |
| Survey administration | Anonymous online questionnaire conducted between January and February 2026. | Methods – Study Design / Participants |
| Voluntary participation | Participation was voluntary and no incentives were provided. | Methods – Participants |
| Response rates | Medical students: 70.5%; Faculty: 17.7%. | Results – Demographic Characteristics |
| Handling incomplete questionnaires | Only complete questionnaires were included in the analyses. | Methods – Participants |
| Prevention of multiple entries | The institutional survey system restricted participation to invited individuals; duplicate responses were not identified. | Methods – Participants |
| Statistical analysis | Descriptive statistics, t tests, χ² tests, ANCOVA, Pearson correlations, multiple linear regression, pooled interaction model with HC3 robust standard errors, and ordinal logistic regression sensitivity analysis. | Methods – Statistical Analysis |
| Items not applicable | Cookies, IP checks, log-file analysis, adaptive questioning, randomization of items, and open public access were not applicable because the survey was conducted through an institutional invitation-based system. | Not applicable |

## Footnote

The survey was conducted through the institutional communication system and was accessible only to invited participants. Participation was voluntary and anonymous. Only complete questionnaires were included in the final analyses. Because the survey was not publicly accessible, several CHERRIES items (eg, cookies, IP address checks, log-file analysis, adaptive questioning, and randomization of items) were not applicable.
